# Heparin Precursors with Reduced Anticoagulant Properties Retain Antiviral and Protective Effects That Potentiate the Efficacy of Sofosbuvir against Zika Virus Infection in Human Neural Progenitor Cells

**DOI:** 10.3390/ph16101385

**Published:** 2023-09-29

**Authors:** Isabel Pagani, Linda Ottoboni, Paola Panina-Bordignon, Gianvito Martino, Guido Poli, Sarah Taylor, Jeremy E. Turnbull, Edwin Yates, Elisa Vicenzi

**Affiliations:** 1 Viral Pathogenesis and Biosafety Unit, Division of Immunology, Transplantation and Infectious Diseases, IRCCS San Raffaele Scientific Institute, 20132 Milan, Italy; pagani.isabel@hsr.it; 2 Neuroimmunology Unit, Division of Neuroscience, IRCCS San Raffaele Scientific Institute, 20132 Milan, Italy; linda.ottoboni@unimi.it (L.O.); panina.paola@hsr.it (P.P.-B.); martino.gianvito@hsr.it (G.M.); 3 School of Medicine, Vita-Salute San Raffaele University, Via Olgettina 58, 20132 Milan, Italy; poli.guido@hsr.it; 4 Human Immuno-Virology Unit, Division of Immunology, Transplantation and Infectious Diseases, IRCCS San Raffaele Scientific Institute, 20132 Milan, Italy; 5 Department of Biochemistry & Systems Biology, ISMIB, University of Liverpool, Liverpool L69 7ZB, UK; s_taylor11@hotmail.co.uk (S.T.); j.e.turnbull@keele.ac.uk (J.E.T.); 6 Department of Life Sciences, Keele University, Keele, Staffs ST5 5BG, UK

**Keywords:** Zika virus, heparin, heparin derivatives, heparin precursor fractions, protective effects, combination therapy

## Abstract

Zika virus (ZIKV) infection during pregnancy can result in severe birth defects, such as microcephaly, as well as a range of other related health complications. Heparin, a clinical-grade anticoagulant, is shown to protect neural progenitor cells from death following ZIKV infection. Although heparin can be safely used during pregnancy, it retains off-target anticoagulant effects if directly employed against ZIKV infection. In this study, we investigated the effects of chemically modified heparin derivatives with reduced anticoagulant activities. These derivatives were used as experimental probes to explore the structure–activity relationships. Precursor fractions of porcine heparin, obtained during the manufacture of conventional pharmaceutical heparin with decreased anticoagulant activities, were also explored. Interestingly, these modified heparin derivatives and precursor fractions not only prevented cell death but also inhibited the ZIKV replication of infected neural progenitor cells grown as neurospheres. These effects were observed regardless of the specific sulfation position or overall charge. Furthermore, the combination of heparin with Sofosbuvir, an antiviral licensed for the treatment of hepatitis C (HCV) that also belongs to the same Flaviviridae family as ZIKV, showed a synergistic effect. This suggested that a combination therapy approach involving heparin precursors and Sofosbuvir could be a potential strategy for the prevention or treatment of ZIKV infections.

## 1. Introduction

Pharmaceutical heparin, which was first identified over 100 years ago [1], has been used in clinical practice for many decades as a primary anticoagulant agent in surgeries and for the treatment of thrombosis [2,3]. Heparin is a linear anionic polysaccharide composed of repeating disaccharide units with various sulfate groups attached. Its structure closely resembles the polysaccharide component found on the cell surface and the extracellular matrix known as heparan sulfate proteoglycans (HSPGs) [4,5,6]. HSPGs are present in almost all metazoan cells and interact with a wide array of signaling molecules, cytokines, and other host proteins; however, they are also exploited by numerous pathogenic microorganisms for cell attachment. Among viruses, examples include the herpes simplex virus (HSV) [7], Ebola [8], and SARS CoV-2 [9,10], as reviewed by Cagno et al. [11], as well as members of the Flaviviridae family: dengue virus (DENV), Japanese encephalitis virus (JEV), yellow fever virus (YFV), tick-born encephalitis virus (TBEV), and Zika virus (ZIKV), as reviewed by Kim et al. [12]. HSPGs, along with other negatively charged glycosaminoglycans, function as low-affinity attachment factors for flaviviruses, facilitating a virus concentration on the cell surface; however, the binding efficiency to HSPGs may vary depending on the specific virus [13]. Mutations in the envelope protein (E protein) of flaviviruses were found to increase their affinity to HSPGs, which, in turn, affects virulence and neuroinvasiveness [14]. In an interesting functional genomic study, it was also shown that the infection of HeLa cells by ZIKV is dependent upon factors involved in HS and heparin sulfation [15].

ZIKV was first identified in 1947, following its isolation from a Rhesus monkey in the Zika (Ziika) Forest, near Entebbe, Uganda [16,17], although its origin can be traced back to as early as 1928 [18]. Sporadic outbreaks of usually relatively mild febrile disease were reported in 2013–2014 in French Polynesia and other Pacific Islands, where ZIKV infection was also associated with an increased prevalence of Guillain-Barré syndrome in adults [19,20]. ZIKV infection came to prominence in Brazil around 2015, along with a sharp increase in serious birth defects, commonly including microcephaly [21,22], and other fetal abnormalities associated with a high risk for developmental disorders at birth or during the first year of life [23,24,25]. Although ZIKV infections are not nearly as prevalent currently as they were at their peak in 2016, documented cases still occur in over 80 countries, with approximately 18,000 infections per year, as reported on 8 February 2021 (https://cdn.who.int/media/docs/default-source/documents/emergencies/zika/zika-epidemiology-update_february-2022_clean-version.pdf?sfvrsn=c4cec7b7_1&download=true, accessed on 12 September 2023). These cases are mostly found in areas close to the equator, as mosquito bites of female, daytime-active, arboreal *Aedes* mosquitoes, principally *A. aegypti* and *A. albopictius,* remain the primary mode for virus transmission [26], although other modes of transmission, including the sexual route [27,28,29] and blood transfusion [30], have been proven.

ZIKV enters host cells by attaching its E protein to HSPGs present on the cell surface [31,32,33] and binding to multiple receptors, including AXL and TIM1 [34,35,36]. Once inside the cell, after the fusion of the viral membrane is mediated by the acidic pH of the endosomes, the ZIKV-positive-stranded genomic RNA of ca. 10,500 nucleotides is translated into one polyprotein in the cytosol. The polyprotein is then cleaved by the virally encoded protease complex NS2B-NS3 to generate structural (S) and non-structural (non-S) proteins that serve to generate new virions after the non-S proteins form the replication complex. The genomic positive-stranded RNA is transcribed by the NS5 replication complex into a negative-stranded RNA acting as a template for producing multiple copies of progeny positive RNAs. These newly synthesized positive RNAs can either be recruited for further rounds of translation/replication or incorporated into virions. Finally, these assembled virions enter the secretory pathway and undergo maturation, whereby mature virions are released into the extracellular space [37].

The main targets of ZIKV infection are human neural progenitor cells (hNPCs), which are highly permissive to infection and viral replication [38,39,40]. While ZIKV can also infect mature neurons, it does so to a lesser extent [38]. The ability to infect and damage progenitor cells is a major factor contributing to ZIKV’s impact on the neurodevelopment of the brain [41]. Studies involving ZIKV infection with pregnant female mice, non-human primates, and humans have confirmed that ZIKV can cause cell death and cerebral cortex disease [42,43]. Furthermore, ZIKV was also shown to infect peripheral neurons and induce cell death in these neurons as well [44,45,46]. Animal studies showed that ZIKV can infect a wide range of organs in a similar manner to that observed in humans. Some of these immune-privileged organs include the eyes [47,48], and the male [49] and female genital tracts [50,51]. Additionally, after the initial infection, the virus can persist for months and be found in different body fluids, such as ocular secretions, urine, and semen [52,53].

To address ZIKV-induced damage, particularly in the developing fetal brain, we hypothesized that heparin, a drug commonly used to prevent thromboembolic complications during pregnancy, could potentially prevent ZIKV infection in hNPCs *in vitro*. Previous research has shown that heparin can inhibit the attachment of the influenza A H5N1 virus [54] and has anti-inflammatory properties [55]. Given these complementary activities and the potential to repurpose heparin, we initiated our original studies to explore its possible application against ZIKV infection [32,56]. While heparin exhibited only weak inhibition of infection, it was capable of fully reversing the cytopathic effects and cell death observed in hNPCs, derived from reprogrammed human-induced pluripotent stem cells (hiPSCs), restoring them to the levels of uninfected controls [56]. More recently, using neurospheres (NS) formed from hNPCs that aggregate into spheres, heparin was found to prevent ZIKV-induced vacuole formation (a characteristic of paraptosis, a non-apoptotic programmed cell death) while also inhibiting cell necrosis and apoptosis [32]. Furthermore, heparin inhibited ZIKV replication in hNPCs infected at a low multiplicity of infection by approximately 2 log_10_ units. Its primary mode of action interferes with interference with virion attachment while maintaining its protective effect against ZIKV-induced cytopathicity and successfully restoring the ability of hNPCs to differentiate into neuro-glial cells [32]. However, any potential application of heparin (Hep) for new therapeutic purposes must take into account its residual anticoagulant activity as a side effect. Therefore, our initial aim was to investigate whether desulfated heparin derivatives, known for considerably diminished anticoagulant properties [31], could provide protection against ZIKV infection, replication, and cell death in NS. Additionally, we explored the potential of precursor fractions obtained during the purification of heparin as a plentiful source of similar protective compounds with reduced anticoagulant activity [57]. These chemically modified derivatives, with systematically varied substitution patterns, are denoted as D1 to D7 [58]. Meanwhile, the precursor fractions previously referred to as A to D in reference [57] will now be referred to as F1 to F4. In addition, heparin and the most active fractions were combined with Sofosbuvir, an antiviral drug clinically used for the treatment of HCV infection [59]. Sofosbuvir is an analog of a uridine nucleotide that acts similarly to the physiological nucleotide, effectively blocking the NS5B polymerase and inhibiting HCV-RNA synthesis by terminating the RNA chain [60]. Previous studies have also demonstrated the inhibitory effects of Sofosbuvir on ZIKV infection [61,62]. Therefore, our hypothesis was that combining heparin with its anti-attachment activity against ZIKV, or its most active precursors, along with Sofosbuvir has the potential to block two crucial steps of the ZIKV cycle: viral entry and viral RNA synthesis. This combination could potentially lead to additive or synergistic antiviral effects.

## 2. Results

Prevention of cell death by chemically modified heparin derivatives and heparin precursor fractions

(i) Chemically modified heparin derivatives (D1 to D7) prevent cell death independently of anticoagulant activity.

**Figure 1:**
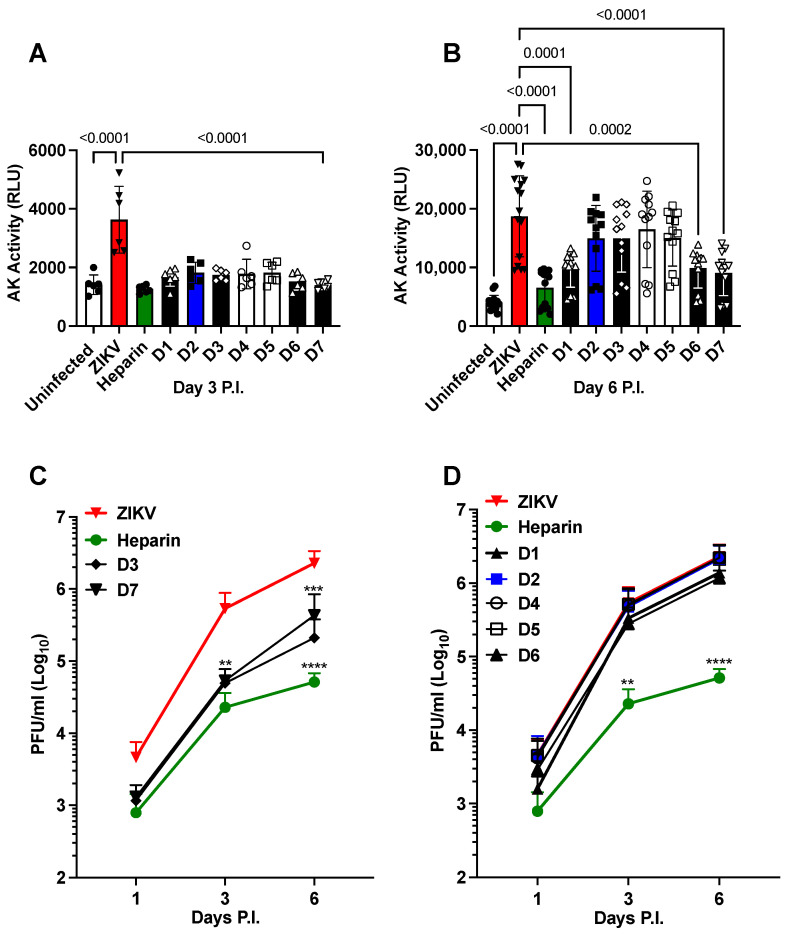
Effects of heparin derivatives on ZIKV infection of the NS. Predominant repeating disaccharides of heparin and chemically modified heparin derivatives denoted as D1–D7 were used. Heparin: IdoA2S-GlcNS6S. D1: Ido2S-GlcNAc6S. D2: Ido2OH-GlcNS 6S. D3: Ido2S-GlcNS6OH. D4: Ido2OH-GlcNAc6S. D5: Ido2S-GlcNAc6OH. D6: Ido2OH-GlcNS6OH. D7: Ido2OH-GlcNAc6OH. NS were pretreated with heparin derivatives (100 µg/mL) 1 h prior to infection with ZIKV (Brazilian 2016-INMI-1 isolate) at a MOI of 1. (**A**) Cell death was measured by adenylate kinase (AK) activity 3 days post-infection (P.I.) and (**B**) after 6 days of P.I. (**C**) Kinetics of viral replication in the presence of D3 and D7 derivatives compared to infected untreated (ZIKV) and heparin-treated NS. (**D**) Kinetics of viral replication in the presence of D1, D2, D4, D5, and D6 derivatives compared to infected-untreated and heparin-treated NS. The bars in panels (**A**,**B**) represent the mean ± standard deviation (SD) of five independent experiments in triplicate. The kinetics of viral replication represent the mean ± SD of two independent experiments in triplicate. The *p* values were calculated using a one-way ANOVA with a Tukey correction for multiple comparisons. ** indicate *p* < 0.01, *** *p* < 0.001, and **** *p* < 0.0001 as calculated by one-way ANOVA with Dunnett’s correction, comparing the mean of each day’s P.I. values to that of ZIKV control.

All tested chemically modified heparin derivatives (D1 to D7), along with unmodified API heparin (heparin), demonstrated significant prevention of cell death in NS when treated with 100 µg/mL at day 3 post-infection (P.I.), without adversely affecting cell viability (Figure 1A). Furthermore, heparin, D1, D6, and D7 exhibited significant reductions in ZIKV-induced cell death at day 6 P.I. (Figure 1B). Importantly, this protective effect is not contingent on the structural features responsible for heparin’s anticoagulant activity, as elucidated in Table 1. Additionally, the overall charge of the derivatives does not exert a significant influence, with the average charge per disaccharide ranging from 3.5 (2.5 sulfates plus 1.0 carboxylate) in heparin to just over one negative charge (0.2 sulfates plus 1 carboxylate) in D7.

Given our prior demonstration of heparin’s ability to inhibit ZIKV infection in NS [32], we sought to determine whether its derivatives could yield similar inhibitory effects. In line with our previous findings, treatment with heparin resulted in a significant reduction in the infectious titers of ZIKV in the culture supernatant, decreasing it by approximately 1.6 log_10_ on day 6 of P.I. Among the tested derivatives, only D3 and D7 exhibited a significant reduction in ZIKV infectious titers at both day 3 and 6 days of P.I., decreasing it by approximately 1 log_10_ (Figure 1C). In contrast, the remaining derivatives did not exhibit any inhibitory effect on viral replication (Figure 1D). These results imply that neither the specific position of sulfation nor the overall negative charges alone can account for the antiviral effects of heparin and its ability to prevent ZIKV-induced cell death in NS.

While these chemically modified heparin derivatives are instrumental for investigating structure-activity relationships and possess significantly reduced anticoagulant potential (all derivatives, D1 to D7, exhibit less than <1% of the anti-factor Xa activity of heparin [58]), they have effectively demonstrated the ability to dissociate anticoagulation from antiviral and cell-protected activities. However, due to limitations in the availability of these modified heparin derivatives, we turned to precursor fractions, F1 to F4, possessing reduced anticoagulant activities and which are typically discarded during the production of API heparin from crude heparin. These fractions contain a variety of structurally diverse polysaccharides and are produced in large quantities. Earlier research has indicated that these fractions retain certain other activities of heparin, such as supporting cell signaling of fibroblast growth factor (FGF2) through the HS-dependent FGF receptor tyrosine kinase system, FGFGR1c [57]. Therefore, we investigated these fractions for their potential antiviral and cell-protective activities against ZIKV infection.

(ii) Heparin precursor fractions (F1 to F4) possess antiviral activities independently of their reduced anticoagulant effects.

**Figure 2:**
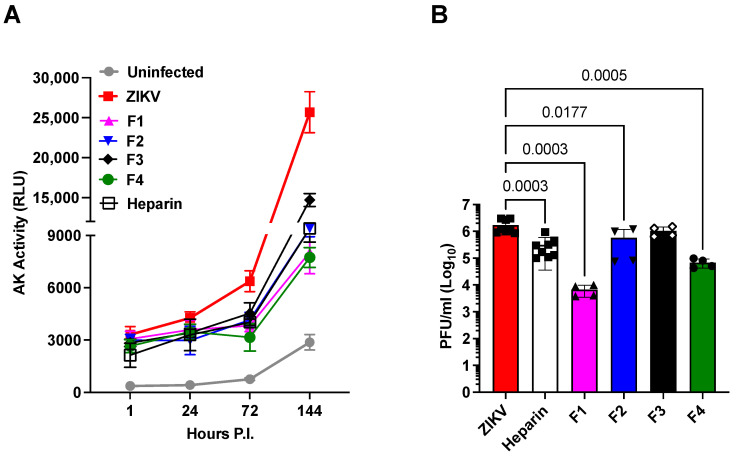
Effects of heparin precursor fractions on the infection of ZIKV in NS. (**A**) Kinetics of cell death induced by ZIKV (Brazilian 2016-INMI-1) at a MOI of 1. Heparin and precursor fractions were tested at 100 µg/mL. Cell death was measured using the activity of AK expressed in relative light units (RLU). (**B**) Effect of the precursor fractions on viral replication measured by plaque-forming units (PFU) at 6 days of P.I. The bars represent the mean ± SD. The experiments were repeated five times for panel A and twice for panel B. *p* values were calculated using a one-way ANOVA with Dunnett’s correction.

The antiviral activities of heparin precursor fractions, F1 to F4, were evaluated by measuring cell death prevention by assaying adenylate kinase (AK) activity released by damaged cells (Figure 2A), and their impact on viral replication was assessed by plaque-forming units (PFU) 6 days after infection (Figure 2B). The precursor fractions were tested at 100 µg/mL, a dosage that did not significantly affect cell viability. All fractions demonstrated significant ability to protect cells against cell death (Figure 2A). However, their effects on viral replication displayed variability; F3 demonstrated minimal impact compared to the untreated control, while F1 reduced viral replication by approximately 30% (Figure 2B). It is noteworthy that the anticoagulant activities of these heparin precursor fractions were significantly diminished. The range of residual activity spanned from 11% of heparin activity for F1 and F3 to the highest residual activity of 37% in the APTT assay for F4 (Table 2). These results confirm that, like heparin derivatives, the anticoagulant properties of heparin precursor fractions can be separated from their antiviral activity.

(iii) The overall charge of the heparin precursor material fractions F1 to F4 does not correlate with their antiviral activities, and no single substitution predominates in the most active fractions, F1 and F4.

The molecular weight (M_w_), dispersity (D), and average sulfation per disaccharide unit (DS) were also considered (Table 1) in relation to the inhibition of ZIKV-induced cell death. To investigate this, NS were incubated with the precursor fractions prior to infection, and we measured the kinetics of ZIKV-induced cell death using the AK activity in the culture supernatant. When tested at 100 µg/mL, all fractions, except F3, inhibited AK activity (Figure 2A) and viral replication to a similar extent as heparin (Figure 2B). Notably, among the fractions, F1 exhibited the highest potency, reducing the plaque-forming units (PFU) by 2.4 log_10_, compared to the 0.85 log_10_ reduction achieved by heparin. Additionally, F4 demonstrated greater potency than heparin, with a reduction of 1.36 log_10_ compared to the 0.85 log_10_ reduction observed with heparin. Interestingly, we observed no straightforward correlation between antiviral activity and the basic properties of charge density or molecular weight. This observation aligns with the complex interactions of heparan sulfate (HS) and heparin derivatives in various biological activities [63]. These findings suggest that interactions with target proteins depend on the structure of the derivative rather than simple charge density. In general, interactions between compounds of this class and proteins may encompass a range of activities at the level of individual polysaccharide chains. Therefore, the observed activity of a particular fraction is a composite result of the activities of numerous chains that constitute that fraction.

(iv) Synergistic antiviral effects were observed for the dual use of Sofosbuvir, an established antiviral agent, and heparin.

**Figure 3:**
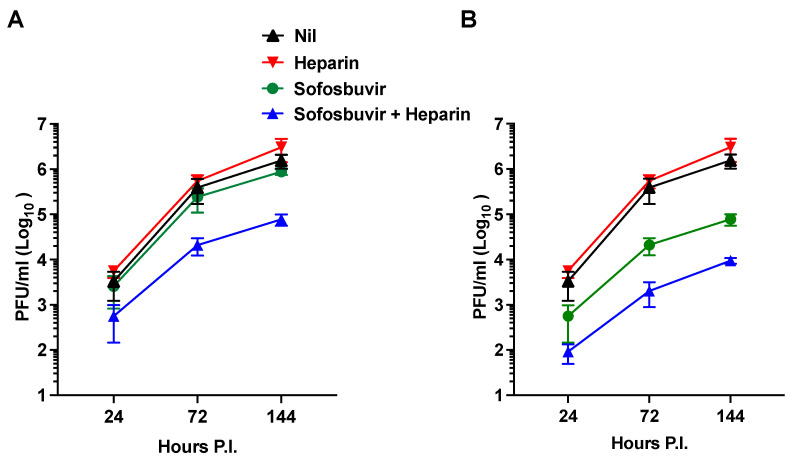
Synergistic effects of the antiviral agent, i.e., Sofosbuvir, and heparin in hNPCs infected with the Puerto Rico 2015-PRVABC59 (CDC) isolate at a MOI of 1. (**A**) Reduction in PFU over a 7-day time course employing the combined application of heparin (100 µg/mL), Sofosbuvir (10 µg/mL), and the two drugs in combination. (**B**) Reduction in PFU over a 7-day time course employing the application of heparin (100 µg/mL), Sofosbuvir (100 µg/mL), and the two drugs in combination (both at 100 µg/mL). The kinetics represent the mean ± SD of three independent experiments in triplicate.

In the past decade, significant advancements have been made in the treatment of hepatitis C caused by the hepacivirus (HCV), a member of the Flaviviridae family. One of the breakthrough treatments is Sofosbuvir, a nucleotide analog inhibitor of viral RNA synthesis [59], which has been recognized as an essential medicine by the WHO. Additionally, Sofosbuvir has demonstrated inhibitory effects on ZIKV infection by targeting viral RNA synthesis [61]. In this study, our aim was to explore the potential advantages of combining Sofosbuvir and heparin as they target distinct steps in the ZIKV life cycle (viral RNA synthesis and entry, respectively). To investigate this, we initially tested two concentrations of Sofosbuvir (10 and 100 µg/mL) and heparin (100 µg/mL) in the infection of hNPCs at a MOI of 1. Figure 3A illustrates that Sofosbuvir alone did not effectively inhibit ZIKV replication at a concentration of 10 µg/mL, and heparin alone exhibited limited antiviral activity. However, when Sofosbuvir was combined with heparin, a synergistic antiviral effect emerged, resulting in an approximate 1.4 log_10_ reduction in viral replication after 7 days of P.I. This synergistic effect persisted when Sofosbuvir was tested at a higher concentration of 100 µg/mL, leading to a reduction in viral replication by approximately 2.5 log_10_ and demonstrating synergy with heparin co-treatment (Figure 3B). These findings suggest that the combined application of Sofosbuvir and heparin may offer an effective approach for treating ZIKV infection.

**Figure 4:**
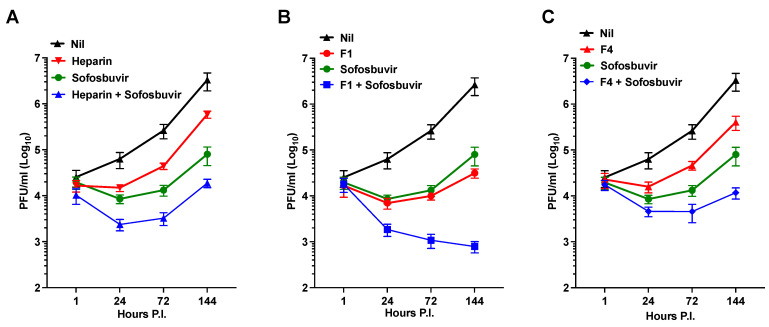
Synergistic antiviral activity of Sofosbuvir with F1 or F4 in NS infected with the Brazilian 2016-INMI-1 isolate at an MOI of 1. (**A**). Kinetics of viral replication using the combined application of heparin (100 µg/mL), Sofosbuvir (100 µg/mL), and both drugs at 100 µg/mL. (**B**) Kinetics of viral replication using the application of F1 (100 µg/mL), Sofosbuvir (100 µg/mL), and both drugs at 100 µg/mL. (**C**). Kinetics of viral replication using the application of F4 (100 µg/mL), Sofosbuvir (100 µg/mL), and both drugs at 100 µg/mL. The kinetics represent the mean ± SD of three independent experiments in duplicate.

Considering the higher efficacy of the F1 and F4 fractions in inhibiting ZIKV infection compared to heparin in hNPCs, we explored the combination of Sofosbuvir with either the F1 or F4 fractions, using heparin as a control, in the infection of NS. Importantly, the co-administration of Sofosbuvir with heparin or its fractions did not exhibit any toxic effects on the cells. Figure 4A demonstrates that Sofosbuvir was more effective than heparin in inhibiting ZIKV replication in NS. However, when combined with heparin, there was a substantial reduction of infectious virus released into the supernatant by 2.2 log_10_ at 144 h P.I. Interestingly, the combination of Sofosbuvir with F1 resulted in a significant reduction in the amount of PFU to 3.5 log_10_ at 144 h P.I. (Figure 4B). In contrast, the effect of F4 was like that of heparin, resulting in a 2.32 log_10_ reduction at 144 h P.I. (Figure 4C).

**Figure 5:**
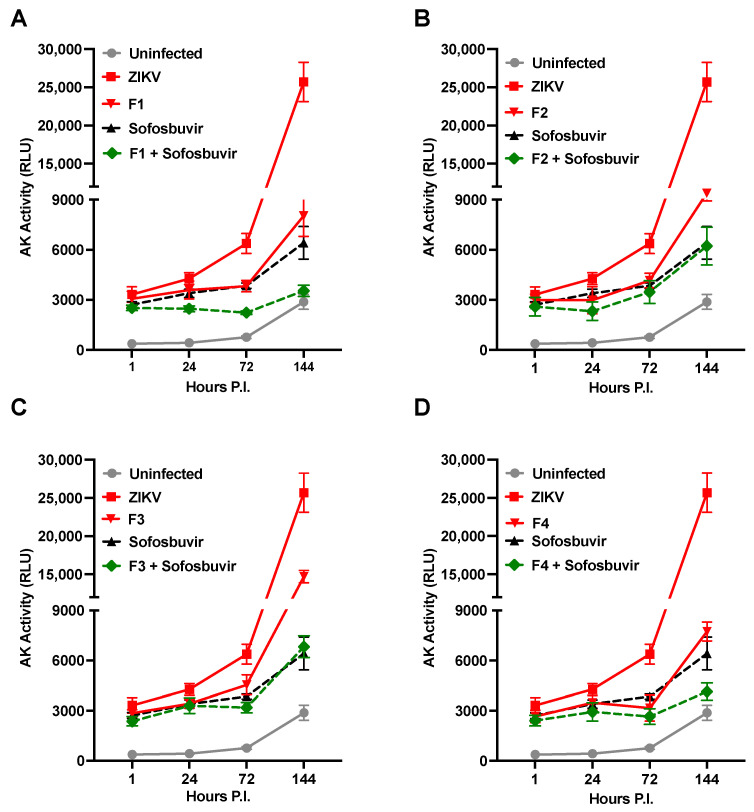
Synergistic anti-cell death activity of Sofosbuvir with F1 or F4 in NS infected with the Brazilian 2016-INMI-1 isolate at an MOI of 1. Kinetics of AK activity with combined application of Sofosbuvir (100 µg/mL) with: (**A**) F1 at 100 µg/mL; (**B**) F2 at 100 µg/mL; (**C**) F3 at 100 µg/mL; and (**D**) F4 at 100 µg/mL. The kinetics represent the mean ± SD of three independent experiments in duplicate.

Moreover, given that precursor fractions of heparin have exhibited the ability to inhibit ZIKV-induced cell death (Figure 2A), we investigated whether the combination of Sofosbuvir with the F1 to F4 fractions could yield a synergistic effect in inhibiting ZIKV-induced cell death. Intriguingly, the combination of Sofosbuvir with the F1 fraction synergistically restored the levels of AK activity to those observed in uninfected cells, as illustrated by the kinetics of AK activity release in the culture supernatant (Figure 5A). Comparable effects were observed for the fraction F4 (Figure 5D), while the F2 and F3 fractions did not exhibit a synergistic effect (Figure 5B,C, respectively). These findings suggest that targeting different steps of the viral life cycle, as has been demonstrated for other viruses like HIV [64], may offer an advantage in controlling viral replication and reducing ZIKV-induced cell death.

## 3. Discussion

Chemically modified derivatives of heparin API and precursor fractions obtained during the manufacturing of heparin API, which exhibit strongly attenuated anticoagulant activity ([58] and Table 1 and Table 2), were found to retain their ability to prevent ZIKV-induced cell death in hNPCs grown in tissue culture as NS (Figure 1 and Figure 2). Moreover, heparin and its precursor fractions demonstrated inhibitory effects on ZIKV infection in hNPCs (Figure 3). Notably, when combined with the drug Sofosbuvir, heparin precursor fractions exhibited a remarkable reduction in viral infection, reducing it by almost 3 log_10_ in hNPC-derived NS (Figure 4).

Heparin’s well-established anticoagulant activity is primarily mediated by its interaction with antithrombin III, a serine protease, which depends on a pentasaccharide sequence within the complex structure of heparin [65,66,67]. However, interactions between heparin and other binding proteins are generally less specific (reviewed by Meneghetti et al. [68]). It is highly likely that this also holds true in this context. The ability of chemically modified heparin derivatives, with their low anticoagulant activities and diverse substitution patterns, to protect against ZIKV infection demonstrates that anticoagulation and antiviral effects can be separated. The results imply that the antiviral activity does not rely on specific sequences within these heparin derivatives. It is interesting that fractions F1 to F4, while exhibiting similar anticoagulant activities (Table 1) as well as broadly similar physico-chemical properties (Table 2), showed more varied activities in ZIKV infection (Figure 2B). The origin of these distinct biological activities is therefore likely to reside in subtle differences in the sequences of structural populations rather than a small number of critical structural features such as sulfate groups at specific positions within the repeating units of the polysaccharides. Variations in bioavailability may also contribute to these differences. Therefore, differences in activity between fractions F1 and F4 are unlikely to result from the presence or absence of a limited number of highly specific sequences.

Effective pharmacological treatment of ZIKV infection is particularly crucial for pregnant women who contract the virus during the first trimester of pregnancy to prevent severe consequences for fetal brain development [38]. Although ZIKV is not currently circulating as it was in 2015–2016 in South America, there remains a risk of its re-emergence. Travelers without prior exposure to ZIKV are susceptible to infection when visiting tropical regions where the virus can spread undetected, as recently reported in Cuba [69]. Heparin and related molecules hold promise as potential candidates for ZIKV treatment. Some forms of heparin are already used safely during pregnancy to treat preeclampsia [70], and chemically modified heparin derivatives have also shown potential [71]. This study explored the use of low-anticoagulant fractions, typically discarded during heparin production, to uncouple anticoagulant activity from the beneficial effects against ZIKV. Despite being generally less sulfated than intact heparin (Table 2), these materials maintain their activity while exhibiting diverse structural variants, sulfation patterns, and sulfation sizes [57,58]. This suggests that, similar to many heparin-interacting proteins, this antiviral activity does not rely on a unique structure or a highly limited set of structural variants.

We chose the NS model of ZIKV infection because hNPCs can aggregate to form 3D structures, simulating neurogenesis, unlike hNPCs grown in the cell monolayer [56]. The NS model allows for the observation of heparin’s antiviral activity under a high multiplicity of infection conditions.

Apart from heparin, several drugs used in clinical practice for other medical purposes have been evaluated for potential anti-ZIKV activity. One such drug is Sofosbuvir, which has demonstrated anti-ZIKV activity [61]. Sofosbuvir is a nucleotide analog inhibitor and a direct-acting antiviral agent that disrupts the HCV life cycle. It is first metabolized into its active form, which acts as a defective substrate for NS5B, an RNA-dependent RNA polymerase, thus limiting virus replication [72]. Notably, when combined with heparin, which has limited antiviral activity on its own, Sofosbuvir exhibits a synergistic effect. This synergy is maintained and strengthened as the Sofosbuvir concentration increases to 100 µg/mL. This suggests that heparin and Sofosbuvir target different pathways in the ZIKV life cycle. Previous research has shown that heparin interferes with the early steps of viral entry by blocking ZIKV attachment to target cells, while Sofosbuvir inhibits a post-entry step of the ZIKV life cycle by targeting the RNA-dependent RNA polymerase, thereby preventing RNA synthesis [32,56].

## 4. Conclusions

The success of combination antiretroviral therapy in treating HIV/AIDS has emphasized the significance of employing drugs that target different stages in a virus’s life cycle. The findings presented in this study indicate that further exploration of the potential of precursor fractions obtained from readily available heparin production could pave the way for enhanced therapies for ZIKV infection. These precursor fractions, currently manufactured in substantial quantities, exhibit promise when combined with the antiviral medication Sofosbuvir. This approach presents a compelling pathway for advancing treatments against ZIKV infection and could offer an appealing route to improved therapies for this condition.

## 5. Materials and Methods

### 5.1. Viruses

The Brazilian 2016-INMI-1 (GenBank Accession # KU991811) was obtained from an Italian individual who traveled to Brazil in January 2016. Puerto Rico 2015-PRVABC59 was obtained from the CDC (GenBank Accession #KU501215). Both viral isolates were cultured in Vero cells and quantified in a plaque-forming assay (PFA), with the procedure outlined below.

### 5.2. Human iPSC-Derived NPCs and Neurospheres (NS)

Fibroblasts were isolated from a skin biopsy of one healthy subject as described [73]. These fibroblasts were then reprogrammed into pluripotent stem cells (iPSCs) using the episomal Sendai virus method (CytoTune-iPS 1.0 Sendai Kit, Life Technologies, Carlsbad, CA, USA) to obtain human iPSCs (hiPSCs). hiPSCs were cultured in feeder-free conditions using mTeSR1 culture medium (STEMCELL Technologies, Vancouver, BC, Canada) on Matrigel ES-coated plates (MaES, Corning, Corning, NY, USA) and passaged using 0.5 mM EDTA. To generate hNPCs, a modified version of the protocol described in Reinhardt et al. [74] was used. Briefly, colonies of hiPSCs grown on MaES were detached using dispase (STEMCELL Technologies) and maintained in human embryonic stem cell (hESC) medium without basic fibroblast growth factor (bFGF). The medium was supplemented with 1 µM dorsomorphin (Stemgent, Beltsville, MD, USA), 3 µM CHIR99021 (Tocris, Bristol, UK), 10 µM SB-431542 (Miltenyi, Bergisch Gladbach, Germany), and 0.5 µM purmorphamine (Alexis). Embryoid bodies (EBs) were formed by culturing cells in non-tissue culture Petri dishes (Greiner, Kremsmunster, Austria). On day 2, the medium was changed to N2B27 medium, which consisted of equal parts Neurobasal (Invitrogen, Waltham, MA, USA) and DMEM-F12 medium (Invitrogen) with a 1:100 B27 supplement lacking vitamin A (Invitrogen) with 1:200 N2 supplement (Invitrogen), 1% penicillin/streptomycin/glutamine (PSG, Gibco, Grand Island, NY, USA), and the same small molecules as used above. On day 4, dorsomorphin and SB-431542 were removed from the medium, and 150 µM ascorbic acid (AA) was added. On day 6, the Ebs were mechanically dissociated into smaller aggregates and seeded onto Matrigel growth factor-reduced high-concentration-coated 12-well plates (Ma GFRH, Corning). When hNPCs reached confluence, they were detached with Accumax (Sigma, St. Louis, MO, USA) and replated (at least 1:5) in the presence of a 0.5 µM ROCK inhibitor (Calbiochem, San Diego, CA, USA). After 3 passages, purmorphamine was substituted with 1 µM SAG (Calbiochem). The hNPCs were cultured until passage 10 before being utilized. To obtain hNPCs grown as NS, single cells were maintained in suspension in non-tissue culture Petri dishes, allowing them to form aggregates.

### 5.3. Infections

hNPCs were cultured under orbital rotation for three days to achieve optimal growth. One hour prior to infection, porcine intestine mucosal heparin (Celsus), heparin derivatives, and fractions were added at a final concentration of 100 µg/mL. The NS were then infected with the Brazilian 2016-INMI-1 isolate at a MOI of 1. Viral supernatants were collected at 3 and 6 days post-infection. To assess cell death, adenylate kinase activity levels were measured in the supernatants using the ToxiLight Bioassay from Lonza. Additionally, the viral titers were determined by a PFA.

hNPCs grown in 2D were initially seeded at a density of 2.5 × 10^5^ cells/well in 24-well flat-bottom plastic plates using 500 μL of complete medium. After 24 h, hNPCs were subjected to pre-treatment with either Sofosbuvir (AstaTech, Inc., Bristol, PA, USA) or heparin and a combination of both. Subsequently, the cells were inoculated with the PRVABC59 isolate at a MOI of 1. Following a 6-day incubation period, viral supernatants were collected. The viral titers were determined using PFA.

### 5.4. Plaque Forming Assay (PFA)

Vero cells were seeded in 6-well culture plates at a density of 1.2 × 10^6^ cells. After 24 h, virus-containing supernatants were ten-fold diluted in culture medium supplemented with 1% heat-inactivated FBS, and 1 mL of each dilution was added to the cells. The plates were incubated for 4 h at 37 °C. After the incubation period, the unabsorbed virus was removed. Next, 2 mL of culture medium supplemented with 1% methylcellulose (Sigma) were added to each well, followed by an incubation at 37 °C for 6 days. Following the incubation period, the methylcellulose overlay was removed, and the cells were stained with 1% crystal violet in 70% methanol. Plaques were then counted and expressed as plaque-forming units per mL (PFU/mL), as previously published [56].

### 5.5. Cell Death Detection Assay

To measure the adenylate kinase (AK) activity in culture supernatants as a marker of cell death, the ToxiLight^®^ BioAssay (Lonza, Norwest, Australia) was utilized. Briefly, culture supernatant samples (10 μL) were transferred onto a black 96-well half-area plate (Costar, Washington, DC, USA). A detection reagent (50 μL) was added to each well, and the plate was incubated for 10 min at room temperature. Luminescence was measured using a Mithras LB940 Microplate Reader (Berthold Technologies, Bad Wildbad, Germany). The results were expressed as relative luminescent units (RLU).

### 5.6. Heparin and Chemically Modified Heparin Derivatives (D1 to D7) and Heparin Precursor Fractions (F1 to F4)

Heparin (derived from porcine intestinal mucosa) was obtained from Celsus. Chemically modified derivatives of heparin (D1 to D7) were prepared following the methods described in reference [58], which involved several chemical de-sulfation and re-N acetylation steps. Their anticoagulant properties and physiochemical characteristics have been described elsewhere [58]. The heparin precursor fractions used in this study were generously provided by Guanzhou Yang Fan, Guangdong, China. The structural characteristics and anticoagulant properties of these fractions have been reported [57]. The anticoagulant properties of fractions F1 to F4, heparin, and heparin derivatives D1 to D7 were measured as described [43].

### 5.7. Anticoagulation Assays

APTT assays. The test samples were assayed against the sixth International Standard for unfractionated heparin using an automated coagulometer (ACL-TOP 550, Werfen Ltd., Warrington, UK). Concentration response curves with at least three dilutions and replicates for both the standard and the test were assayed. Potency estimates and the statistical analysis of the data were carried out in accordance with European Pharmacopoeia guidelines, using CombiStats5.0 (European Directorate of the Quality of Medicine (EDQM)). The specific activity in IU/mg was calculated from the potency obtained by each assay method relative to the dry weight of the test sample. Briefly, human plasma (normal) was obtained from whole blood post-collection (into sodium citrate; 11 mM final concentration) by centrifugation (1500 *g*, 15 min). Test samples were prepared in tris-buffered saline (TRIS 50 mM, NaCl 150 mM, pH 7.4), and each dilution was mixed with plasma before the addition of Pathrombin SL (100 μL) reagent. After incubation, clotting was started by the addition of calcium chloride (50 μL, 50 mM, 37 °C), and the clotting time was recorded.

Anti-Xa and anti-IIa assays. The methods used were modified from the method described in the United States Pharmacopoeia (USP) general monograph for the assay of heparin, adapted for use on an automated coagulometer. Relative anticoagulation potential was determined using a clinical-grade Coatest heparin test kit (Chromogenix) that was previously adapted to a 96-well plate format, reading absorbance at λ = 405nm (Multi Scan EX plate reader, Thermo Fisher Scientific, Waltham, MA, USA).

Heparin cofactor II assays. Samples were diluted with tris-buffered saline and mixed 1:1 with heparin cofactor II (2.5 μg/mL). Human thrombin (1 IU/mL) was added to the mixture and incubated for 420 s prior to the addition of a chromogenic substrate specific for thrombin (S2238, 1 mM). The change in color was recorded at 405 nm.

### 5.8. Weight Average, Molecular Weight, and Dispersity

Briefly, weight average molecular weight (M_w_) and dispersity (M_w_/M_n_) were determined by gel permeation chromatography on an Agilent GPC 50 system (Agilent, Santa Clara, CA, USA) equipped with TSK G3000 and G2000 (7.8 mm × 30 cm) columns in series with refractive index detection and reference to the USP heparin sodium molecular weight calibrant (as a broad range molecular weight calibrant).

### 5.9. Degree of Sulfation

Samples were solubilized in heparinase buffer to make a solution of 10 mg/mL. 200 µg of by-products were digested with 2.5 mU of heparinase III, I, and II (IBEX) added sequentially at 2-hour intervals, with heparinase II incubating at 37 °C overnight. Further digestion with fresh aliquots of all three heparinases was performed for 2 h to ensure samples were digested fully; no evidence of significant polymetric material was found by gel electrophoresis. The products of digestion were separated according to charge on a strong anion exchange Propac PA1 column (4 mm × 250 mm, Dionex, Sunnyvale, CA, USA), eluted with a linear gradient of 0–2M NaCl, over 60 min at 1 mL/min. Product peaks were detected by UV at 232 nm, and samples were compared to 8 disaccharide standards of known structure (Dextra Labs, Reading, UK), also separated on the same column and under the same conditions. The average number of sulfate groups per repeating disaccharide was calculated from the disaccharide compositional analysis.

## Figures and Tables

**Table 1:** Anticoagulant activity (AT anti-factor X_a_) and degree of sulfation (DS) for intact heparin (Hep) and heparin derivatives D1 to D7 [58].

	AT Anti-X_a_ (%Hep) ^a^	DS ^b^
Hep	100	2.50
D1	0.03	1.68
D2	0.40	1.92
D3	0.50	1.26
D4	0.03	1.05
D5	0.03	0.46
D6	0.03	0.85
D7	0.03	0.20

^a^ Percent of anti-factor X_a_ activity relative to intact heparin (100%) specific activity (IU/mg); ^b^ Degree of sulfation: average number of sulfate groups per repeating disaccharide.

**Table 2:** Anticoagulant activities (APTT, AT anti-IIa, AT anti-X_a_, and HCII-anti-factor X_a_), molecular weight (M_w_), dispersity (DP), and degree of sulfation (DS) for heparin precursor fractions F1 to F4 [57].

	APTT ^a^	AT Anti-IIa ^b^	AT Anti-Xa ^c^	HCII-Anti-Xa ^d^	M_w_^e^	D ^f^	DS ^g^
F1	11	6	11	6	30.6	2.2	1.8
F2	34	27	28	23	18.7	2.0	2.1
F3	11	9	11	7	33.5	2.1	1.6
F4	37	35	37	3	26.3	1.7	2.0

^a^ Plasma antithrombin-dependent activated partial thromboplastin clotting time (s) as a percentage of sixth Int. stnd; 209 s. ^b^ Percentage antithrombin-dependent anti-factor II_a_ activity relative to Hep (100%) specific activity in IU/mg. ^c^ Percentage antithrombin-dependent anti-factor X_a_ activity relative to Hep (100%) specific activity in IU/mg. ^d^ Percentage heparin cofactor II-dependent anti-Xa activity relative to Hep (100%) measured as specific activity in IU/mg. ^e^ Weight average molecular weight determined by gel permeation chromatography (Agilent GPC 50). ^f^ Dispersity (M_w_/M_n_). ^g^ Degree of sulfation: average number of sulfate groups per repeating disaccharide calculated from the disaccharide compositional analysis following complete digestion with heparinase I, II, and III and quantification of the resulting disaccharide separation (HPAEC) compared to reference standards.

## Data Availability

Data is contained within the article.
